# The Characteristics of Radial Growth and Ecological Response of *Caragana korshinskii* Kom. Under Different Precipitation Gradient in the Western Loess Plateau, China

**DOI:** 10.3389/fpls.2022.862529

**Published:** 2022-04-08

**Authors:** Cunwei Che, Shengchun Xiao, Aijun Ding, Xiaomei Peng, Jingrong Su

**Affiliations:** ^1^Key Laboratory of Ecohydrology of Inland River Basin, Northwest Institute of Eco-Environment and Resources, Chinese Academy of Sciences, Lanzhou, China; ^2^University of Chinese Academy of Sciences, Beijing, China; ^3^Gansu Agricultural University, Lanzhou, China; ^4^Key Laboratory of Desert and Desertification, Northwest Institute of Eco-Environment and Resources, Chinese Academy of Sciences, Lanzhou, China

**Keywords:** *Caragana korshinskii* Kom, plantations, radial growth, planting density, precipitation

## Abstract

Understanding the temporal-spatial variability of tree radial growth and ecological response is the basis for assessing forest vulnerability in sight of climate change. We studied stands of the shrub *Caragana korshinskii* Kom. at four sampling sites (natural forest CL and plantation forests XZJ, CK and TPX) that spanned the different precipitation gradient (180–415 mm) across China’s western Loess Plateau, and demonstrated its radial growth dynamics and ecological response. We found that the growth of natural *C. korshinskii* in arid regions have adapted and cope with regional environmental changes and radial growth was less affected by drought stress. However, the growth of planted *C. korshinskii* was significantly affected by drought stress in arid and semi-arid regions, especially during the growing season (from June to September). Variations in radial growth rates and growth indicators such as shrub height, canopy area are consistent with the climate-growth relationship. With increase of precipitation, the limiting of drought on the growth of planted *C. korshinskii* gradually decreased and the amount of radial growth variation explained by drought decreased from 53.8 to 34.2% and 22.3% from 270 to 399 and 415 mm of precipitation, respectively. The age-related radial growth trend shows that radial growth increased until 4 years of age, then decreased rapidly until 12–14 years of age, and then eventually tend to stabilized. In the context of climate warming and humidification, increased precipitation and regular branch coppicing management at around 12 years old will help to mitigate the limitation of drought on the growth of *C. korshinskii*. Moreover, the initial planting density should be tailored to local precipitation conditions (below 5,000 shrubs per hectare). The above results have important practical significance for the maintenance of the stability and sustainable management of plantation forests in the western Loess Plateau.

## Introduction

Understanding the temporal-spatial variability of tree radial growth and ecological response is the basis for assessing forest vulnerability in sight of climate change (Venegas-González et al., 2018; Wang et al., 2019, 2020). Because of their single species and structure, plantation forests are expected to be more vulnerable to climate change than natural forests (Navarro-Cerrillo et al., 2019; Santini et al., 2020; Camarero et al., 2021). The Loess Plateau has experienced long-term climate changes and human activities, resulting in the destruction of natural forests, and severe soil erosion (Feng et al., 2016; Li et al., 2016). In response to the above problems, the Chinese government started to implement vegetation restorations such as Grain for Green Project (GFGP) in 1999, which aim to halt desertification in the northwest China, and alleviate soil erosion (Li et al., 2021). As a consequence, soil erosion has decreased by 24% and vegetation cover has increased by 6–8% (Jia and Shao, 2013; Jiang et al., 2016). The normalized difference vegetation index (NDVI) data show that the mean annual NDVI increased from 0.454 to 0.613 in the Loess Plateau during 1999–2013 (Gao et al., 2017).

Afforestation efforts, however, are not always successful due to a lack of understanding about the suitability of planted species to the local environments and their ecological responses (Zastrow, 2019; Li et al., 2021). This has led to severe soil desiccation in deep soil layer of the Loess Plateau’s arid and semi-arid ecosystems, resulting in high mortality rates of plantation forests (Cao, 2008; Fu et al., 2011; Liu and Shao, 2015; Li et al., 2021). Recent studies have shown that, if afforestation managers can control planting density and maintain a program of regular branch coppicing, plants are less affected by declines in soil moisture; vegetation degradation can be improved (Xiao et al., 2015; Navarro-Cerrillo et al., 2019; Li et al., 2021). The selection of the time of branch coppicing was determined by the measurements of soil water content (SWC) (Cheng et al., 2009, Cheng X. R. et al., 2013; Jia and Shao, 2013). However, these studies were carried out over short-time period, we do not know how the interventions studies would fare over the long term. If we are to manage plantation forests properly, we need to study plant’s ecological response on longer time scale.

Dendroecology is dependable, well-replicated technique for reveal climate-growth relationships and ecological responses on a longer time scale (Bar et al., 2008; Hallinger et al., 2010; Liang et al., 2015). Shrubs and dwarf shrubs offer a potential opportunity to extend the dendrochronological networks in extreme climate regions. For example, at high latitudes regions (Sturm et al., 2001; Blok et al., 2011; Le Moullec et al., 2019), arid and semi-arid regions (Srur and Villalba, 2009; Copenheaver et al., 2010; Xiao et al., 2019), and high altitudes regions (Barichivich et al., 2008; Lu et al., 2022). These studies also indicate a promising research direction of shrub-growth relationships (Lu and Liang, 2013).

*Caragana korshinskii* Kom. is the dominant shrub species in semi-desert regions. In northwest China, natural *C. korshinskii* is mainly found in the Alxa Desert, and has been widely introduced planting to the Loess Plateau due to its strong sand-fixing and soil and water conservation functioning (Li et al., 2006). In the previous study, Xiao et al. (2015) found that the semi-shady slope of Beishan in Lanzhou was the most suitable habitat for the growth of *C. korshinskii*. However, this study focused on the same area with different slope aspects and did not analyze the radial growth under different precipitation gradients, which may be more important for plantation forest growth in water-limited Loess Plateau region.

For this reason, we used dendroecology method to analyze the characteristics of radial growth and ecological response of *C. korshinskii* under different precipitation gradients in the western Loess Plateau. We also tested the hypothesis that variations in radial growth and ecological response differ between natural and planted shrubs *C. korshinskii* and may be affected by differences in precipitation and shrubs density. We aimed to (i) clarify the radial growth and main limiting factor of shrubs growth under different precipitation gradient; (ii) determine the suitable age of branch coppicing in the growth process and most suitable afforestation region in the western Loess Plateau.

## Materials and Methods

### Study Area and Species

**FIGURE 1:**
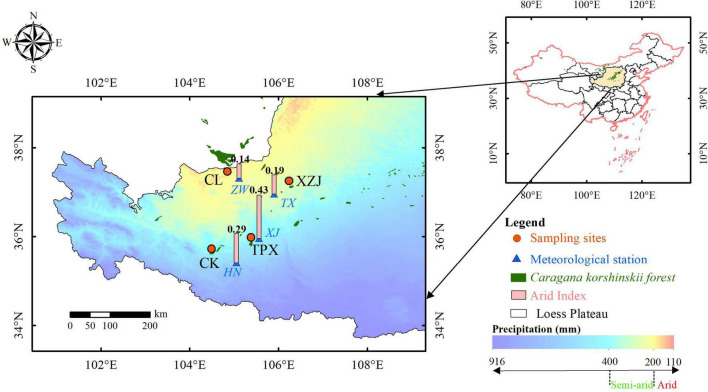
Map of the sampling sites and nearest meteorological stations.

The study area is located in the western part of Loess Plateau and bordering the eastern part of Alxa Desert. It has a typical temperate continent arid climate: the annual mean temperature is 7–9°C, the annual mean precipitation is 185–450 mm, precipitation mainly occurs from June to September, generally in the form of torrential rains (Gao et al., 2017). The most common soil types are loessal soil and sierozem developed on the parent material of quaternary eolian loess. In the southeast, the area is best described as temperate arid steppe, and gradually transitioning to desert steppe and steppe desert in the northwest, vegetation cover is consistent with the precipitation pattern, and gradually improves from northwest to southeast (He, 2021; Zhang et al., 2021; Figure 1).

*Caragana korshinskii* is a legume and the genus is Caragana. It is naturally distributed in desert regions such as Alxa Desert, western Ningxia, and areas in the Gansu Hexi Corridor (Lu et al., 2012; Figure 1). Because of its stress tolerance and utility in controlling shifting sands, it has been widely used in reforestation and afforestation projects (Li et al., 2006; Fang et al., 2007; Xiao et al., 2015).

Total four sampling sites were chosen: ChangLiu (37°27′N, 104°50′E, 1,638 m), henceforth CL; XinZhuangJi (37°15′N, 106°13′E, 1,630 m), henceforth XZJ; Chankou (35°43′N, 104°28′E, 2,147 m), henceforth CK, and TianPingXiang (35°58′N, 105°22′E, 2,028 m), henceforth TPX; Where CL is natural shrub stand and others are plantation stands. The geographic location of these sites, main habitat characteristics, and the spatial distribution of precipitation are shown in Figure 1 and Table 1. Based on the magnitude of aridity index (AI), which is the ratio of annual precipitation to potential evapotranspiration (PET). There are four dryland subtypes defined by the AI: hyper-arid (AI < 0.05), arid (0.05 ≤ AI < 0.20), semi-arid (0.20 ≤ AI < 0.50), and dry sub-humid (0.50 ≤ AI) regions (Middleton and Thomas, 1997). According to this definition, our sampling sites were divided into two climate regions, that is the arid region (CL and XZJ) and the semi-arid region (CK and TPX).

**TABLE 1:** Environmental characteristics of each sampling site.

Sampling sites	Geographic location	Habitat	Landscape
CL	37°27′N 104°28′E 1,638 m	Located in the southern edge of the Tengger Desert. Annual precipitation is 185 mm, aeolian sandy soil. It is scattered and widely distributed natural stands, lower stand density and usually mixed with *Hedysarum scoparium*.	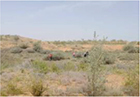
XZJ	37°15′N 106°13′E 1,630 m	Located in the mountain diluvial fan margin. Annual precipitation is 270 mm, sierozem. Afforestation by the row-belt methods and at a density of ∼6,600 shrubs per hectare, and mixed with small herbaceous plants such as *Stipa sareptana*.	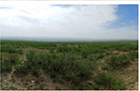
CK	35°43′N 104°28′E 2,147 m	Located in Yinwa mountain. Annual precipitation is 399 mm, loessal soil. Afforestation by the horizontal ditch methods and at a density of ∼5,000 shrubs per hectare. It is mixed with small herbaceous plants, such as *Heteropappus hispidus*.	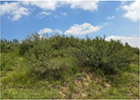
TPX	35°59′N 105°23′E 2,028 m	Located in Xiji county. Annual precipitation is 415 mm, dark loessal (Heilu) soil. Afforestation by the horizontal ditch methods and at a density of ∼3,300 shrubs per hectare, and mixed with small herbaceous plants, such as *Agropyron cristatum*.	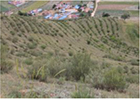

### Sample Collection and Experimental Analysis

Our sampling was conducted in June, (TPX); September, 2020 (CL and XZJ), and July, 2021 (CK). At each site, we collected sample disks of *C. korshinskii.* We selected 20–30 healthy shrubs with no shrub scars for sampling. The sample disks were taken to a laboratory and air-dried before being planned to 3 cm depth disks. They were then sanded with 400-mesh and 800-mesh sandpaper until the tree ring boundaries were clearly visible under the microscope.

Extreme care was taken to ensure dating accuracy. A line was randomly selected for preliminary cross-dating; another line, perpendicular to the first, was also cross-dated. Ring widths were measured to the nearest 0.01 mm along the largest and smallest radius, using the LINTAB ring-width measurement system^1^. After all sample measurements were completed, measured ring widths, and cross-dating results were analyzed as per the COFECHA program.

More than 30 *C. korshinskii* shrubs were chosen randomly for *in situ* measurement. We measured shrub height (ground to tip of the tallest branch), ground diameter (measured at the root collar using a Vernier caliper), canopy area, and the number of branches at each sampling site.

### Tree-Ring Chronologies

The ARSTAN program (Cook, 1985) was used to detrend and standardize our ring-width series. Because each sample disk was measured to the pith, we used a regional curve standardization method (Briffa et al., 1992; Xiao et al., 2015) for detrending. We divided the measured ring-width values by the fitting value after fitting a regional growth curve. This gave us with a standardized ring-width index series for each sampling site (Cook, 1985). We could verify in each set of samples depending on the results of cross-dated: CL 2003–2020, XZJ 2003–2020, CK 1993–2020, and TPX 1999–2020. We were then able to correlate our ring-width chronology with temperature and precipitation records from local meteorological stations using the DendroClim2002 (Biondi and Waikul, 2004) procedure. We took into account lagged effects (lag between meteorology and tree growth) and used meteorological parameters from September of the previous year to September of the current year in our correlations (Gao et al., 2019).

To clearly determine the limiting time of *C. korshinskii* radial growth, we applied the R package “*climwin*” to quantify the climate-growth relationship. The “*climwin*” uses an information-theoretic approach to select the optimal model (with the lowest AICc and highest R^2^) by minimizing the corrected Akaike information criterion (AICc) (Burnham and Anderson, 2002; Bailey and Martijn, 2016; Van et al., 2016). This is more precise and objective compared to the conventional correlation analysis method (Camarero and Alvaro, 2020).

### Climatic Data

Climatic data were collected from the four meteorological stations nearest to the sampling sites (China Meteorological Data Network^2^): Zhongwei (37°32′N, 105°11′E, 1,225 m), Tongxin (36°58′N, 105°54′E, 1,339 m), Huining (35°41′N, 105°05′E, 2,012 m), and Xiji (35°58′N, 105°43′E, 1,916 m). We used the data from these sites (monthly mean temperature and monthly total precipitation data) to analyze the climate-growth relationships (Supplementary Figure 1).

The Standardized Precipitation Evapotranspiration Index (SPEI) combines precipitation and evapotranspiration (ET_0_) data (Vicente-Serrano et al., 2010a; Beguería et al., 2014). We chose this index because SPEI considers multiscale characteristics supporting the identification of different drought types and effects in the context of climate change (Vicente-Serrano et al., 2010b). In addition, the drought response of various hydrological systems (including soil moisture, snow water, and river discharge) to precipitation can significantly change over time (Vicente-Serrano et al., 2010b). Hence, we calculated the 1 month scale SPEI to analyze the response of shrubs to drought in our study. The R package “spei” was used to calculate this index for each meteorological station. Moreover, we calculated variations of radial growth in the 2 years before drought year (PreDr) and the drought year (Dr) (SPEI < 0). Lloret et al. (2011) defined this variation as the ability of trees to resistant drought maintain growth.

### Soil Water Content

We use a soil auger to obtain soil samples. We tried to sample to a depth of 200 cm (because the auger hit bedrock at 150 cm at XZJ sites, we were only able to obtain samples at 150 cm maximum depth). We took samples from the top 50 cm at two levels, 0–20 cm and 20–50 cm. At depths of 50–200 cm, we collected samples at intervals of 50 cm. We took three samples at each level, measured SWC, and averaged it to get the average SWC at that level. In order to explore the influence of branch coppicing on SWC, we collected soil samples at XZJ site from areas where *C. korshinskii* had been coppiced and areas where Un-coppiced was done, samples were collected in September 2020. In addition, we collected soil samples in June and October 2020 in TPX to compare the variation of SWC in the growing water deficiency period (June) and the defoliation water surplus period (October) of *C. korshinskii*. The weight of the wet soil samples was measured by an electronic balance (0.01 g) immediately after collection. The soil samples were then taken to our laboratory, where they were dried to a constant weight in a drying oven at 105 ± 2°C. We compared the dry weight to the wet weight to obtain the SWC at the time of collection.

### Data Analysis

We used one-way analysis of variance (ANOVA) to compare the differences of growth indicators at each sampling site. Using SPSS and ArcGIS for analysis and mapping.

## Results

### Growth Characteristics of *C. korshinskii*

#### Growth Characteristics of *C. korshinskii* Ring-Width

**FIGURE 2:**
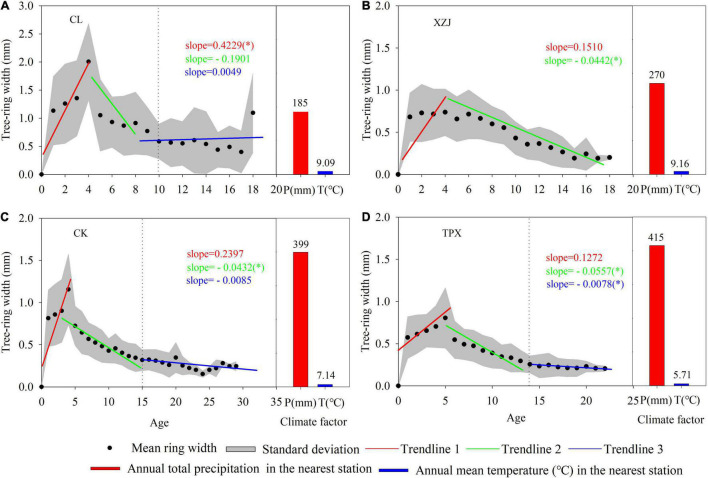
Scatter plots of *C. korshinskii* radial growth corresponding to the tree age and segmented fits as well as climate graphs at each sampling site. Each linear dynamic slope is marked in the same color as the corresponding trendline (Significance: *p < 0.05). **(A)** CL, **(B)** XZJ, **(C)**, CK, and **(D)** TPX.

The results of segmented fit revealing that the radial growth rate of each site *C. korshinskii* increased until an age of 4–5 years (Figure 2), where the natural *C. korshinskii* CL and planted *C. korshinskii* ZXJ in arid regions (mean annual precipitation are 185 and 270 mm, respectively), the radial growth rate was 1.43 and 0.71 mm.yr^–1^, with slopes of 0.42 and 0.15 (Figures 2A,B); while the planted *C. korshinskii* CK and TPX in semi-arid regions (mean annual precipitation are 399 and 415 mm, respectively), the radial growth rates were 0.93 and 0.88 mm.yr^–1^, and with slopes of 0.23 and 0.12, respectively. The smaller slopes indicate slower radial growth rates. At mid-age (6–14 years) period, the growth rate of *C. korshinskii* decreased gradually. In arid regions, natural forest CL was 0.91 mm.yr^–1^ from 4 to 10 years and a slope of −0.19 (Figure 2A); plantation forest XZJ showed a slower decrease after 4 years, with an growth rate of 0.41 mm.yr^–1^ and a slope of −0.04 (Figure 2B). Moreover, the radial growth trend at CK and TPX were similar in semi-arid regions (Figures 2C,D), both gradually decrease at mid-age (0.46 and 0.16 mm.yr^–1^, respectively) and stabilization at around 14 years.

#### Comparison of *C. korshinskii* Growth Parameters

**FIGURE 3:**
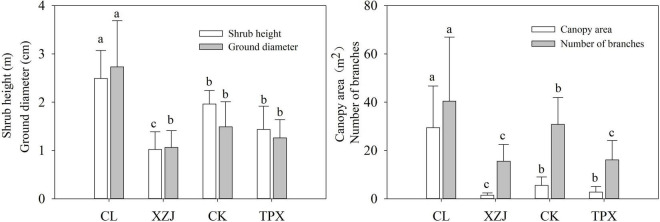
Shrub height, ground diameter, canopy area, and number of branches. Values shown are mean ± SD. Bars for a given parameter are labeled with different letters representing reach significance level at each sampling site (one-way ANOVA, *p* < 0.05).

Shrub height, ground diameter, canopy area, and number of branches are significantly (*p* < 0.05) higher at natural stands (CL) in arid regions than the plantation stands XZJ, CK, and TPX in arid and semi-arid regions (Figure 3). Moreover, all plantation stands sites, the shrub height, and canopy area at XZJ in arid regions were significantly (*p* < 0.05) lower than those at CK and TPX in semi-arid regions, while the latter two sites were not significantly difference in any of the growth indicators, except for the number of branches, which was significantly (*p* < 0.05) higher at CK than TPX (Figure 3).

#### Characteristics of Ring-Width Chronologies

**FIGURE 4:**
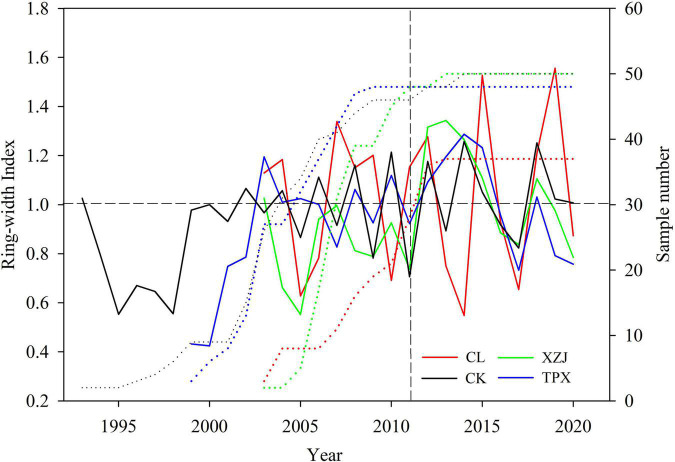
The ring-width chronologies at the four sampling sites after detrending (solid lines represents ring-width index, dotted lines represent sample number).

The mean sensitivities of ring-width chronologies were fluctuating from 0.303 at TPX to 0.542 at CL. There is clearly a high signal-to-noise ratio (SNR) for the results from each of the sites (Table 2). The expressed population signals (EPS) for each of sites were much larger than 0.85. Moreover, the correlations of ring-width chronologies between different sites show that expect for natural forest CL site, all the plantations sampling sites reaches significant correlation of 0.05 (Table 3), illustrating that ring width chronologies of shrubs *C. korshinskii* in different sampling sites all recorded regional environmental information (Figure 4).

**TABLE 2:** The characteristics of ring-width chronologies at each sampling site.

Sampling sites	Radii (disks)	Chronology period	Mean serial correlation	MS	SD	EPS	SNR
CL	37 (27)	2003–2020	0.598	0.542	0.210	0.927	12.738
XZJ	50 (27)	2003–2020	0.527	0.361	0.177	0.928	12.856
CK	50 (28)	1993–2020	0.500	0.340	0.194	0.914	10.663
TPX	48 (28)	1999–2020	0.512	0.303	0.171	0.914	10.573

*MS, mean sensitivity; SD, standard deviation; EPS, expressed population signal; SNR, signal-to-noise ratio.*

**TABLE 3:** Correlation coefficients between four ring-width chronologies (from 2003 to 2020).

Ring-width chronologies	CL	XZJ	CK
XZJ	0.08		
CK	−0.01	0.44*	
TPX	−0.13	0.52*	0.46*

Significance: *p < 0.05.

#### *Caragana korshinskii* Radial Growth and Their Response to Climate

**FIGURE 5:**
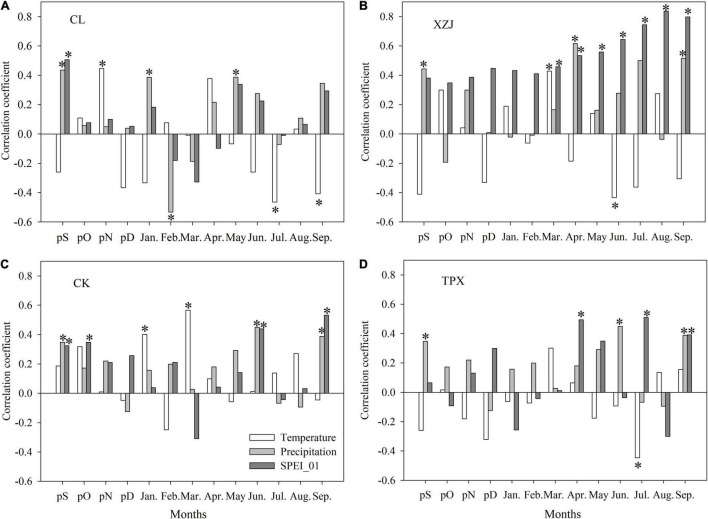
Correlation coefficient between ring-width chronologies and month mean temperature, month total precipitation and SPEI_01 from September of the previous year to September of the current year (Significance: *p < 0.05). **(A)** CL, **(B)** XZJ, **(C)** CK, and **(D)** TPX.

In XZJ and CK sampling sites, shrub *C. korshinskii* radial growth significantly (*p* < 0.05) positively correlated to the temperature in March (Figures 5B,C). However, there was a significantly (*p* < 0.05) negative correlation with temperature during growing season months in CL, XZJ, and TPX, namely, July and September (CL), June (XZJ), and July (TPX) (Figures 5A,B,D).

In all sampling sites, *C. korshinskii* ring width chronology showed a significantly positive correlation with the precipitation in September of the previous year (Figure 5), indicating a lag effect of the precipitation on *C. korshinskii* radial growth. In addition, positive correlations were significant (*p* < 0.05) in the early growing-season (April) in XZJ (Figure 5B). Moreover, during the growing season, we also found a significantly (*p* < 0.05) positive correlation with precipitation, namely, May (CL), September (XZJ, CK and TPX), and June (CK and TPX) (Figure 5).

During the growing season, negative correlations with temperature and positive correlations with precipitation indicated that *C. korshinskii* radial growth was dominantly limited by drought stress. This could also be verified by the relationship between ring-width chronology and SPEI_01 (Figure 5). We found that ring-width chronology and SPEI_01 have significantly (*p* < 0.05) positive correlation in all sampling sites. At CL and CK sites, significantly positive correlations were found in September of the previous year (Figure 5A), this is also indicating a lag effect of the drought stress on *C. korshinskii* radial growth; At XZJ site in arid regions, *C. korshinskii* growth were significantly (*p* < 0.05) positive correlation with SPEI_01 during March–September (Figure 5B). In addition, significantly (*p* < 0.05) positive correlation was detected in June and September at CK in semi-arid regions (Figure 5C). In TPX site, where has more precipitation than other sites, the effect of drought stress only in April, July, and September (Figure 5D).

#### Spatial Contributions of Drought to *C. korshinskii* Radial Growth

The natural forest CL in arid regions, the radial growth of *C. korshinskii* was less affected by drought stress, and the amount of radial growth variability (R^2^) explained by drought only 12.4% (Supplementary Figure 2 and Table 4). However, the radial growth of planted *C. korshinskii* were significantly limited by drought stress in arid and semi-arid regions, and the intensity of limitation gradually decreased with the increase of precipitation at each site (Supplementary Figures 3–5), among which the growth of *C. korshinskii* at TPX with 415 mm precipitation did not reach the significance level of 0.05 with SPEI (Table 4). In addition, the XZJ and CK sampling sites, where precipitation was less compared to TPX (Supplementary Figure 1). The maximum amount of radial growth variability explained by drought corresponded to May–October in the case of XZJ site (*R*^2^ = 0.637), followed by May–June in the case of CK site (*R*^2^ = 0.352), and the minimum amount explained by drought corresponded to February–May in the case of TPX site (*R*^2^ = 0.296). Moreover, the times of climate window determined by “*climwin*” are consistent with the results of the Pearson correlation analysis (Figure 5 and Table 4), illustrating the method is reliable on dendroecology. When we assessed the significance of fitted linear models with K-fold cross-validation and randomization, drought model in the case of XZJ and CK sites still significant, explaining 53.8% and 34.2% of growth variability, respectively (see the histogram in Supplementary Figures 3, 4). However, natural forest CL in arid regions and plantation forest TPX in semi-arid regions did not reach the significance level of 0.05 after cross-validation (Supplementary Figures 2, 5), and the amount of growth variability explained by drought was lower than XZJ and CK sites, at only 12.4% in CL and 22.3% in TPX (Table 4). These results illustrate that the plantation forests with insufficient precipitation is more sensitive to drought stress than the natural forests and plantation forests with sufficient precipitation in the western Loess Plateau.

**TABLE 4:** Climate-growth relationship based on the “*climwin*” model.

Sampling sites	Climate variable	Linear model			Linear model using K-fold cross-variation and randomization method		
		Climate window	ΔAICc	R^2^	Climate Window	ΔAICc	R^2^
CL	Temperature	July–September	−2.71	0.268	August–October	−2.19	0.164
	Precipitation	February–March	−2.92	0.276	January–July	−3.10	0.151
	SPEI_01	April–October	−1.88	0.233	May–September	−3.07	0.124
XZJ	Temperature	June–July	−1.39	0.212	May–July	−2.39	0.093
	Precipitation	February–July	−10.08	**0.514**	March–April	−4.00	**0.354**
	SPEI_01	May–October	−15.33	**0.637**	July–September	−3.59	**0.538**
CK	Temperature	March–April	−6.72	0.280	March–April	−3.12	0.280
	Precipitation	May–June	−8.54	**0.326**	May–June	−3.73	**0.326**
	SPEI_01	May–June	−9.65	**0.352**	May–September	−2.61	**0.342**
TPX	Temperature	June–July	0.24	0.105	May–August	−3.07	0.150
	Precipitation	March–April	−4.01	0.273	August–October	−3.17	0.071
	SPEI_01	February–May	−5.04	0.296	February–July	−3.75	0.223

Significant (p < 0.05) R^2^ values are shown in bold characters. AIC, Akaike information criterion.

In addition, we used CK (399 mm) and TPX (415 mm) with similar precipitation as an example to analyze the effect of planting density on radial growth of shrubs. In drought year 2011, the variation amplitude of ring width index (RWI) was greater in CK than TPX (Figure 4). Moreover, the capacity of resistant drought in CK sampling site (0.70) with higher planting density (5,000 shrubs per hectare) were weaker than TPX sampling site (0.90) with lower planting density (3,300 shrubs per hectare).

## Discussion

### Drought as the Main Driving Factor of Spatial Variability on the *C. korshinskii* Radial Growth

Natural *C. korshinskii* in arid regions have able to adapt to local environment and cope with climate change during their long-term growth process. However, the planted *C. korshinskii* in arid and semi-arid regions, growth was significantly limited by precipitation and drought stress, and the correlations at XZJ in arid regions were higher than CK and TPX in semi-arid regions (Table 4). In addition, the interannual variability of SPEI_01 at the nearest TX meteorological station shows that the variation is larger than the HN and XJ stations (Supplementary Figure 6), together with the lower regional precipitation and higher temperature (Supplementary Figure 1), results in a significant correlation between radial growth and SPEI_01 from March to September at XZJ in arid regions (Figure 5B). While CK and TPX sites in semi-arid regions, where precipitation was more abundant and thus, less affected by drought stress than XZJ (Figures 5C,D). The climate-growth relationship presented above is consistent with the phenological characteristics of natural *C. korshinskii*, which are usually more suitable for growth on shady or semi-shady slopes, where shrubs reduce evaporation to retain soil moisture, allowing for rapidly growth when precipitation is sufficient (Cheng X. R. et al., 2013; Xiao et al., 2015). At the same time, the lower temperature at the TPX sampling site may reduce soil evaporation and retain higher moisture conditions (Figure 2D), which is more conducive to mitigate the effects of drought stress on growth of *C. korshinskii* (Figure 5, Supplementary Figure 5, and Table 4).

### Reason Why Contributions of Temperature, Precipitation, and Drought Stress to *C. korshinskii* Radial Growth Varied at Different Sites

We found a significantly positive correlation between *C. korshinskii* growth and temperature in March at XZJ in arid regions and CK in semi-arid regions (Figures 5B,C), when high temperatures can promote early budding of *C. korshinskii*. In addition, the radial growth of *C. korshinskii* at XZJ and TPX were significantly negatively correlated with temperature and significantly positively correlated with SPEI_01 in June–July (Figures 5B,D), high temperatures occurring in the growing season can increase plant transpiration and soil evaporation, and this greatly increases drought stress. As leaf stomata close and photosynthesis declines, the plant’s ability to synthesize organic matter decreases. It must, however, consume nutrients through respiration. Our finding is also consistent as noted by Zheng et al. (2008). Moreover, we found that with the increase of precipitation, the limiting of drought stress on growth of *C. korshinskii* gradually decreases, and the “*climwin*” model showing that the amount of radial growth variation explained by drought decreased from 53.8 to 34.2% and 22.3% for XZJ, CK, and TPX, respectively (Supplementary Figures 3–5 and Table 4). The reason is that better water conditions during the growing season could increase the accumulation of photosynthetic product and promote the growth of early wood cells of *C. korshinskii*. Hence, we see a significantly positive correlation between growth and precipitation during the growing season (Figure 5).

**FIGURE 6:**
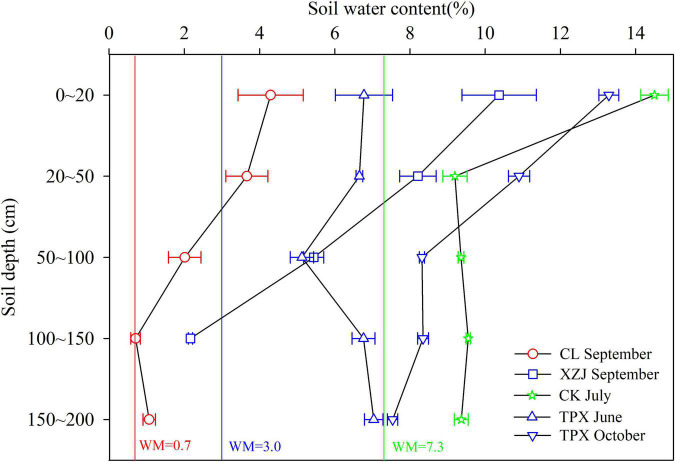
Soil water content at different soil depths for each sampling site (The error bar indicates standard deviation; Red and Green lines show the wilting moisture for sites CL and CK, respectively; Blue line shows the wilting moisture for sites XZJ and TPX).

In the water-limited arid and semi-arid regions, when the SWC is within a range of bounded by wilting moisture (WM) and field capacity (Zhou et al., 2005), it can be utilized by plants. In our study, WM of each site are shown in Figure 6 (Zhou, 2014; Yan et al., 2020). We found that the SWC of natural *C. korshinskii* is higher than WM in arid regions (Figure 6), thus supporting normal growth of shrubs, even though the lower precipitation in this region. However, planted *C. korshinskii* at XZJ in arid regions, the SWC at the 100–150 cm depth was lower than the WM, which is unavailable for shrubs growth. Moreover, *C. korshinskii* need to absorb water at deeper soil layer (>140 cm) during growth season (Li, 2020). Therefore, XZJ was more sensitive to drought stress than the drier CL sites (Figure 5). The SWC of CK and TPX in semi-arid regions were higher than XZJ in arid regions, which may support the normal growth of *C. korshinskii*. In addition, both the SWC in growing water deficiency period (June) and defoliation water surplus period (October) were significantly higher than WM at TPX site, this could further explain that *C. korshinskii* at TPX are less affected by drought stress than XZJ.

During the process of plant growth, planting density is also one of the main factors affecting the response of trees to drought stress (Feng et al., 2016; Navarro-Cerrillo et al., 2019; Li et al., 2021). Table 1 showing the different habitat and planting density of this study. Natural forest CL in arid regions, where *C. korshinskii* is scattered distributed with lower stand density (less than 1,000 shrubs per shrubs), even though low precipitation, the WM was low and SWC enough to support the growth of *C. korshinskii*. Moreover, the growth indicators such as shrubs height and canopy area were significantly higher than those of the plantation forests in semi-arid regions (Figure 3). Therefore, shrubs growth was less affected by drought stress (Figure 5 and Supplementary Figure 2). However, the plantation forests sites under different precipitation conditions, the XZJ planting density was larger (6,600 shrubs per hectare) in arid regions, the area also supported *Stipa sareptana* and other herbaceous plants (Table 1). Together with low precipitation, SWC in the 100–150 cm depth was lower than the wilting moisture. The combined effect of these processes makes the stands of *C. korshinskii* at this area proved to be extremely sensitive to drought stress during growing season (Figure 5B). Site CK and TPX in semi-arid regions received more precipitation than does XZJ in arid regions. It was also planted more sparsely (5,000 and 3,300 shrubs per hectare, respectively). The understory supported only small herbaceous plants such as *Agropyron cristatum* (Table 1). The precipitation in these regions can satisfy the demands of shrub growth (Supplementary Figure 1), thus only sensitive to drought stress in the special months of growing season. Moreover, in different drought years, the capacity of resistant drought in CK sampling site with higher planting density (5,000 shrubs per hectare) were weaker than TPX sampling site with lower planting density (3,300 shrubs per hectare). This result can further support that planting density may modulate the growth response to drought (Xiao et al., 2015; Li et al., 2021).

To address the above questions, we can reduce appropriately planting density and regular coppicing to *C. korshinskii* plantations, which can prevent excessive depletion of soil moisture. Based on the present results, an initial density of around 5,000 shrubs per hectare may be sustainable in the longer term (Li and Guo, 2011; Xiao et al., 2015). In addition, the radial growth rates of planted *C. korshinskii* was faster at the young-age (1–5 years) at each site (Figure 2), while at the middle-age (6–15 years), owing to the increase of shrubs height and canopy area at this time, this caused intense water consumption between adjacent trees, the precipitation was could not meet the demands of shrub growth for soil moisture (Zhao et al., 2005), hence the radial growth gradually decreased at around 12–14 years (Figure 2). This result is also in line with previous findings that the growth indicator of *C. korshinskii* reaching a peak value at middle-age and then gradually decreases and stabilizes in the Loess Plateau (Cheng X. R. et al., 2013). Moreover, the SWC was higher in the coppiced region than the Un-coppiced region (Supplementary Figure 7). Therefore, we may regular coppicing to plantations at middle-age (around 12–14 years) to alleviate the competition between adjacent trees and prevent excessive depletion of soil moisture (Zhao et al., 2005; Cheng X. R. et al., 2013; Xiao et al., 2015).

### Implications for the Management of Plantation Forests in the Context of Climate Warming and Humidification

In this study, *C. korshinskii* at each site were rain-fed without artificial irrigation, precipitation was the main source of the soil moisture supply, and thus, the effective management of plantation forests needs to be reflected in the effective use of precipitation. In the context of climate warming and humidification, precipitation in the Loess Plateau increased at a rate of 7.84 mm⋅yr^–2^, from 2000 to 2015 (Zhang et al., 2021), and the increase in precipitation implies an increase in the available rainfall resources. We can develop technologies for efficient use of rainwater resources to alleviate the conflict between water supply and demand due to drought stress and vegetation restoration. In addition, branch coppicing at the age of 12–14 years is efficient for retaining soil moisture of *C. korshinskii* plantations, especially in semi-arid regions where precipitation is less than 400 mm. Coppicing methods should be adapted to local conditions, such as alternate implement within a single row or clustering at a time (Li and Guo, 2011; Xiao et al., 2015). However, peak radial growth is likely to occur at different ages on sites due to dissimilarities habitats conditions and management strategies (Cheng et al., 2009). Consequently, it is necessary to “adaptation to local conditions” and to determine suitable age for coppicing in different environmental conditions. Moreover, the inverted lance-shaped leaflet characteristics of *C. korshinskii* are more conducive to conservation of soil moisture (Song et al., 2020). Since, large-scale vegetation restoration significantly greened the Loess Plateau and promoted the formation of local precipitation (Feng et al., 2016; Li et al., 2016; Yu et al., 2020; Zhang et al., 2021), the effective management of *C. korshinskii* plantations will help to maintenance the stability of plantations, as well as high-quality development of the Loess Plateau.

## Conclusion

This study demonstrates the characteristics of radial growth and ecological response of shrub *C. korshinskii* under different precipitation gradients in the western Loess Plateau. In arid regions, the natural *C. korshinskii* have adapted and cope with regional environmental changes and radial growth was less affected by drought stress, despite low precipitation in this region. However, the radial growth of planted *C. korshinskii* were significantly limited by precipitation and drought stress in arid and semi-arid regions, especially during the growing season, and with increase of precipitation, the limiting of drought on the growth of *C. korshinskii* gradually decreased, and the variations of radial growth rates and growth indicator such as shrub height, canopy area are consistent with the climate-growth relationship. The greater the precipitation, the more suitable the planting density, the better the growth of *C. korshinskii.* We also note that branch coppicing will effectively retain soil moisture. In order to protect environmental beneficial of *C. korshinskii* plantation stands, managers should plant judiciously and branch coppicing shrubs around 12 years to mitigated excessive depletion of soil moisture. Our results have important theoretical and practical significance for the maintenance of stability and sustainable management of plantations in the context of climate warming and humidification in the western Loess Plateau.

## Data Availability Statement

The original contributions presented in the study are included in the article/Supplementary Material, further inquiries can be directed to the corresponding author/s.

## Author Contributions

CC and SX designed the study. CC analyzed the data and wrote the first version of manuscript. AD, XP, and JS revised the manuscript and approved the submitted version. All authors contributed to the article and approved the submitted version.

## Conflict of Interest

The authors declare that the research was conducted in the absence of any commercial or financial relationships that could be construed as a potential conflict of interest.

## Publisher’s Note

All claims expressed in this article are solely those of the authors and do not necessarily represent those of their affiliated organizations, or those of the publisher, the editors and the reviewers. Any product that may be evaluated in this article, or claim that may be made by its manufacturer, is not guaranteed or endorsed by the publisher.

## Footnotes

1
http://www.rinntech.de

2
http://data.cma.cn
