# Supplementary material for: The Characteristics of Radial Growth and Ecological Response of Caragana korshinskii Kom. Under Different Precipitation Gradient in the Western Loess Plateau, China
Source: Front Plant Sci. 2022 Apr 8;13:862529. doi: 10.3389/fpls.2022.862529 (PMC9024371; doi:10.3389/fpls.2022.862529)
Supplement: Supplementary file 1 [file Table_1.DOC]

**Supplementary Material**

**The characteristics of radial growth and ecological response of *Caragana korshinskii* Kom*.* under different precipitation gradient in the western Loess Plateau, China**

Cunwei Che^a,b^, Shengchun Xiaoa[[1]](#footnote-2), Aijun Ding^c^, Xiaomei Peng^d^, Jingrong Su^a,b^

*^a^ Key Laboratory of Ecohydrology of Inland River Basin,* *Northwest Institute of Eco-Environment and Resources*, Chinese Academy of Sciences, Lanzhou 730000, China

^b^ *University of Chinese Academy of Sciences, Beijing 100049, China*

^c^ Gansu Agricultural University, Lanzhou 730070, China

^d^ Key Laboratory of Desert and Desertification, Northwest Institute of Eco-Environment and Resources, Chinese Academy of Sciences, Lanzhou 730000, China

**Total pages: 7 including cover pages.**

**Total figures: 6**

**S1 Monthly mean temperature and total precipitation at Zhongwei (1959–2020), Tongxin (1955–2020), Huining (1956–2020) and Xiji meteorological stations (1957–2020).**


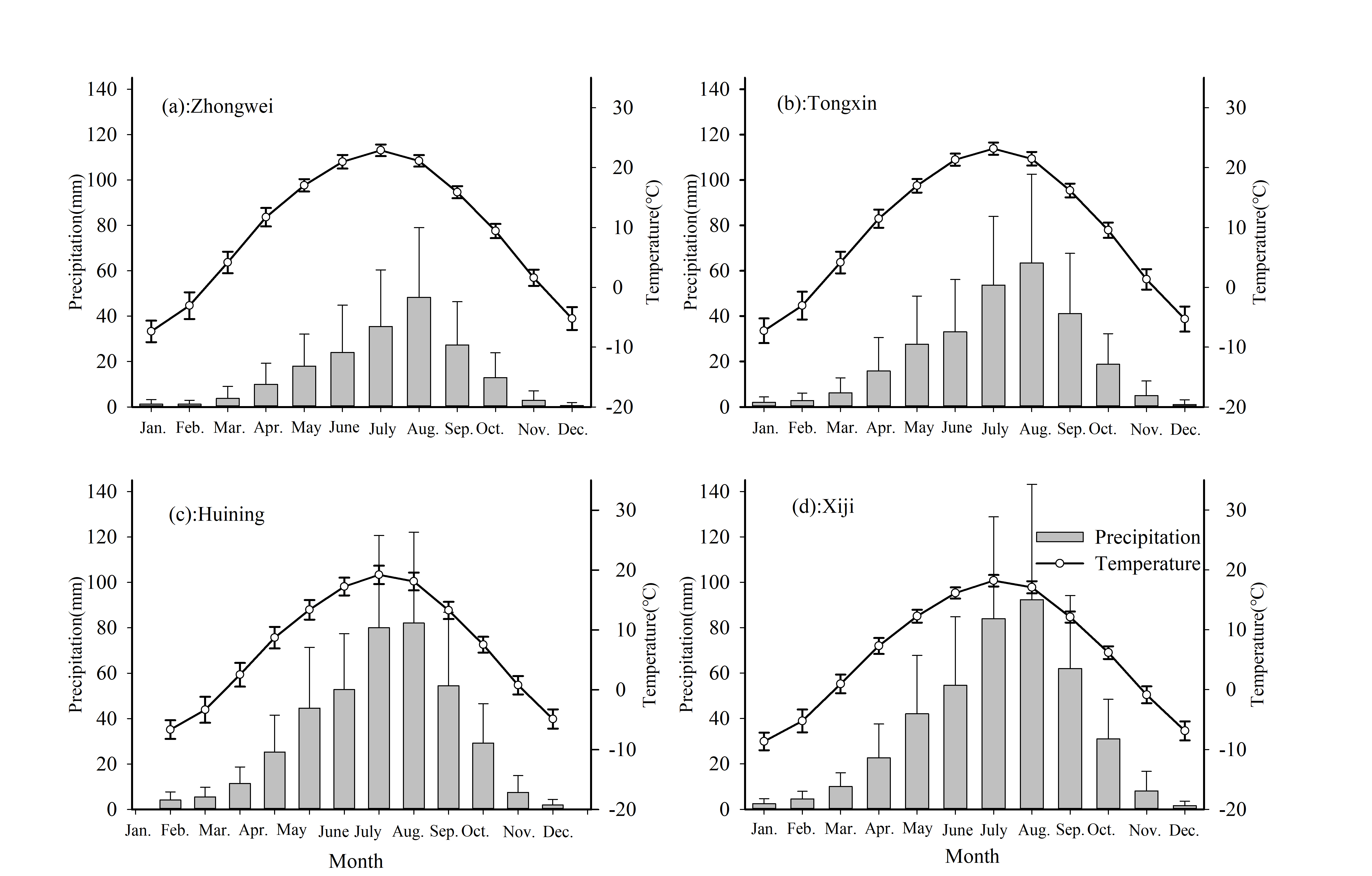


**Fig. S1.** Monthly mean temperature and total precipitation at Zhongwei (1959–2020), Tongxin (1955–2020), Huining (1956–2020) and Xiji meteorological stations (1957–2020).

**S2 Response correlation of CL ring-width chronology and month resolved SPEI: “*climwin*” output panels obtained by calculating with K-fold cross-validation and randomization method (in the histogram of ΔAICc panel the vertical dashed line shows the ΔAICc of the best model fitted on the observed data).**


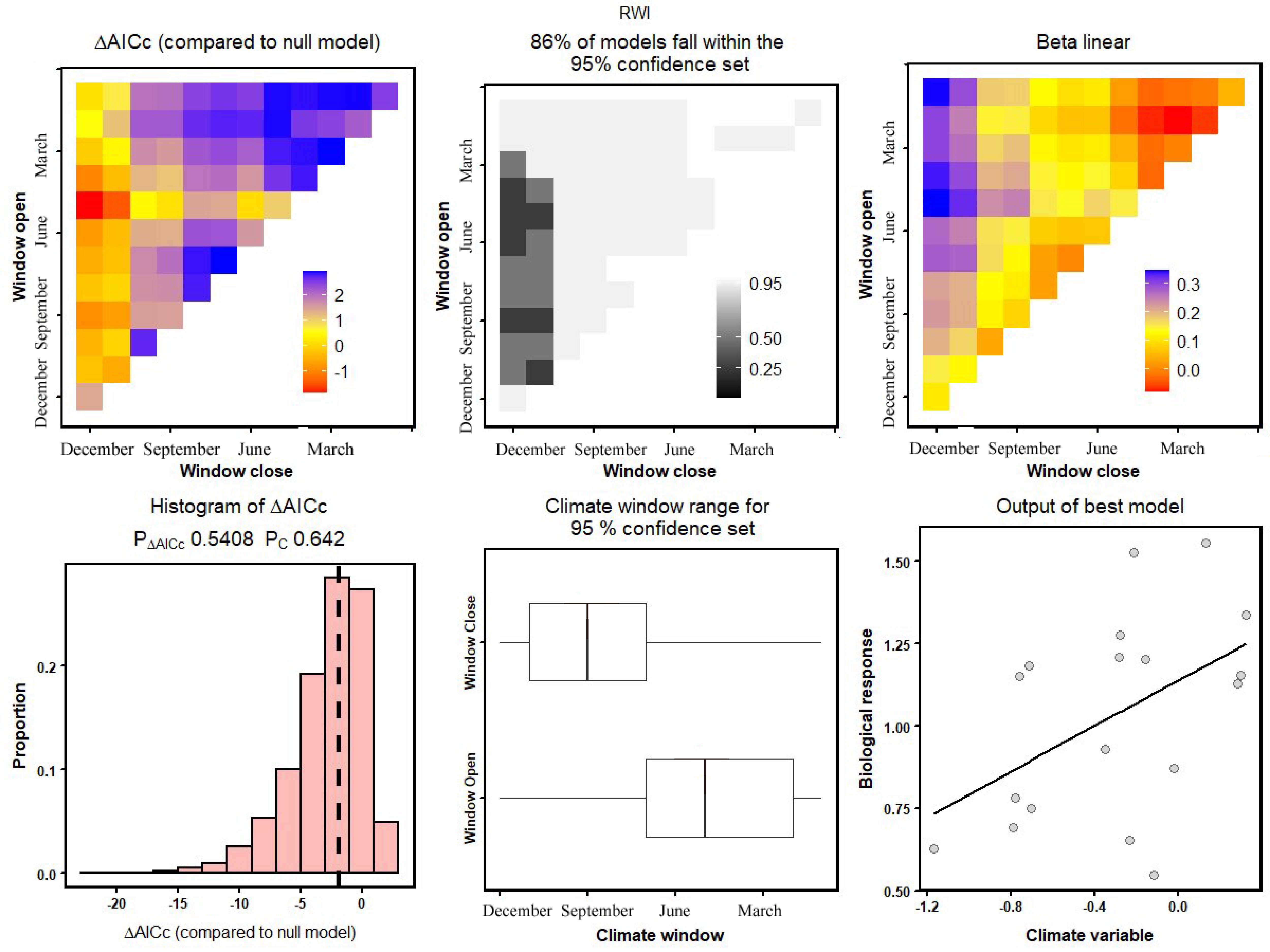


**Fig. S2.** Response correlation of CL ring-width chronology and month resolved SPEI: “*climwin*” output panels obtained by calculating with K-fold cross-validation and randomization method (in the histogram of ΔAICc panel the vertical dashed line shows the ΔAICc of the best model fitted on the observed data).

**S3 Response correlation of XZJ ring-width chronology and month resolved SPEI: “*climwin*” output panels obtained by calculating with K-fold cross-validation and randomization method (in the histogram of ΔAICc panel the vertical dashed line shows the ΔAICc of the best model fitted on the observed data).**


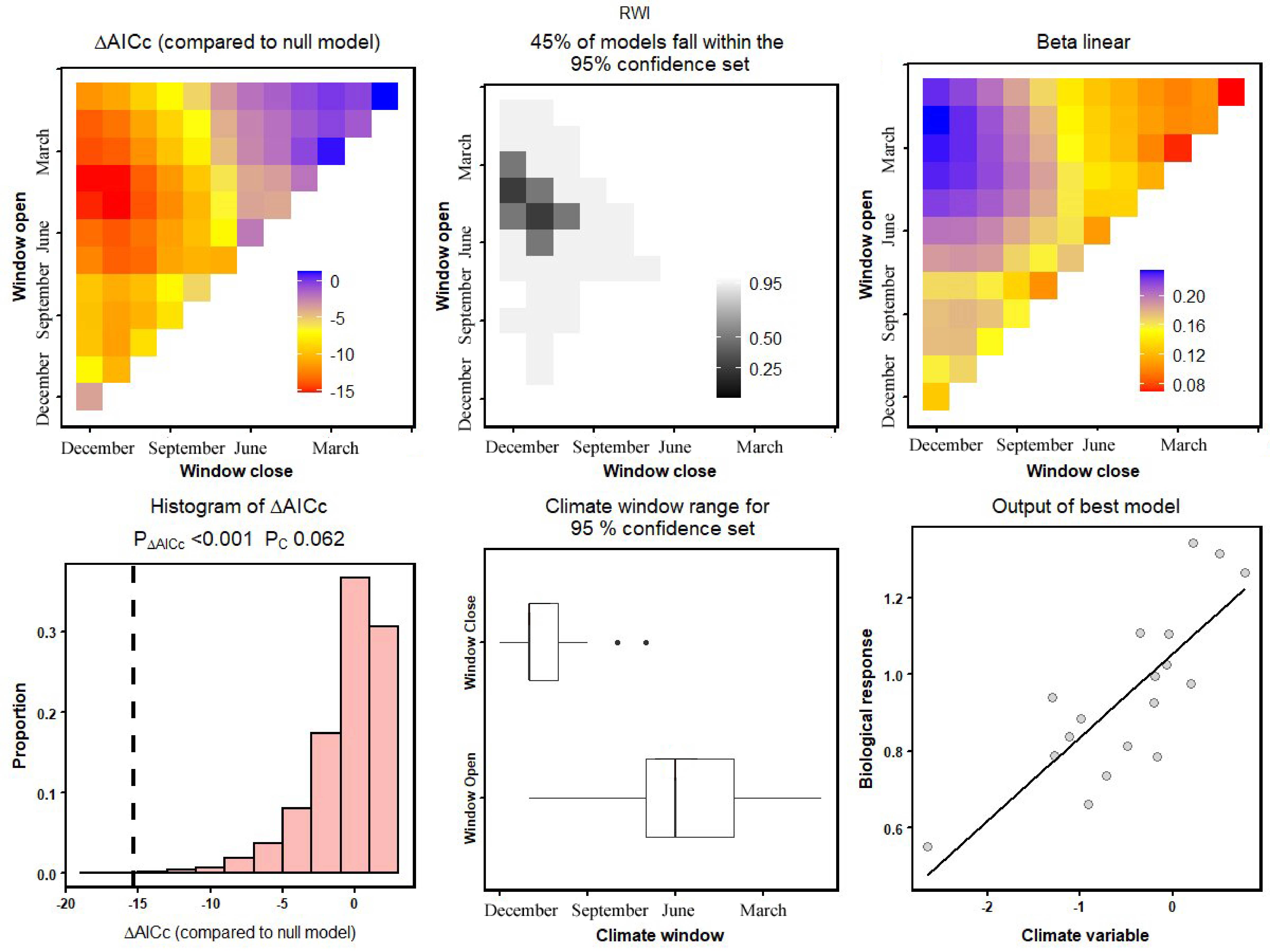


**Fig. S3.** Response correlation of XZJ ring-width chronology and month resolved SPEI: “*climwin*” output panels obtained by calculating with K-fold cross-validation and randomization method (in the histogram of ΔAICc panel the vertical dashed line shows the ΔAICc of the best model fitted on the observed data).

**S4 Response correlation of CK ring-width chronology and month resolved SPEI: “*climwin*” output panels obtained by calculating with K-fold cross-validation and randomization method (in the histogram of ΔAICc panel the vertical dashed line shows the ΔAICc of the best model fitted on the observed data).**


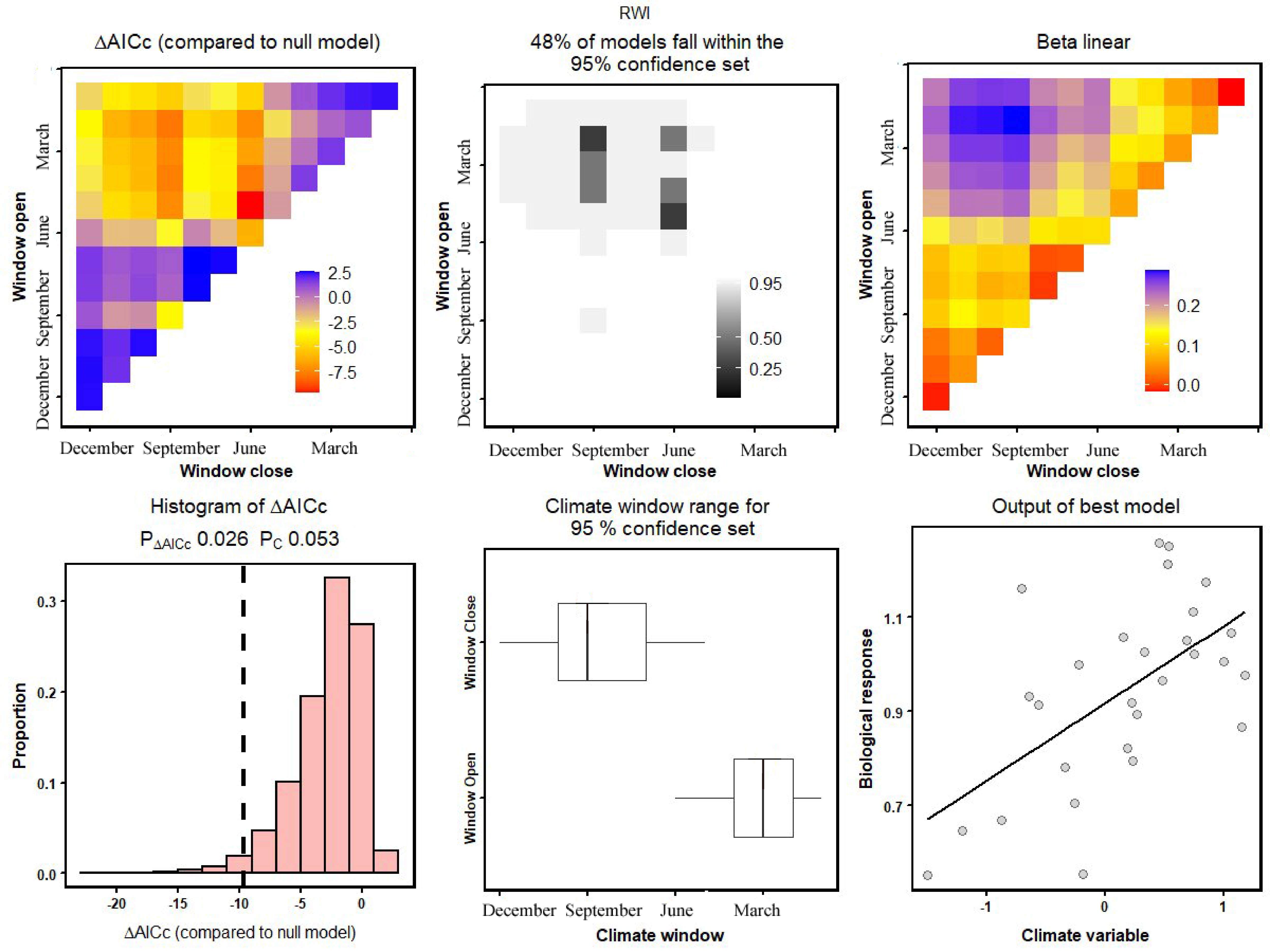


**Fig. S4.** Response correlation of CK ring-width chronology and month resolved SPEI: “*climwin*” output panels obtained by calculating with K-fold cross-validation and randomization method (in the histogram of ΔAICc panel the vertical dashed line shows the ΔAICc of the best model fitted on the observed data).

**S5 Response correlation of TPX ring-width chronology and month resolved SPEI: “*climwin*” output panels obtained by calculating with K-fold cross-validation and randomization method (in the histogram of ΔAICc panel the vertical dashed line shows the ΔAICc of the best model fitted on the observed data).**


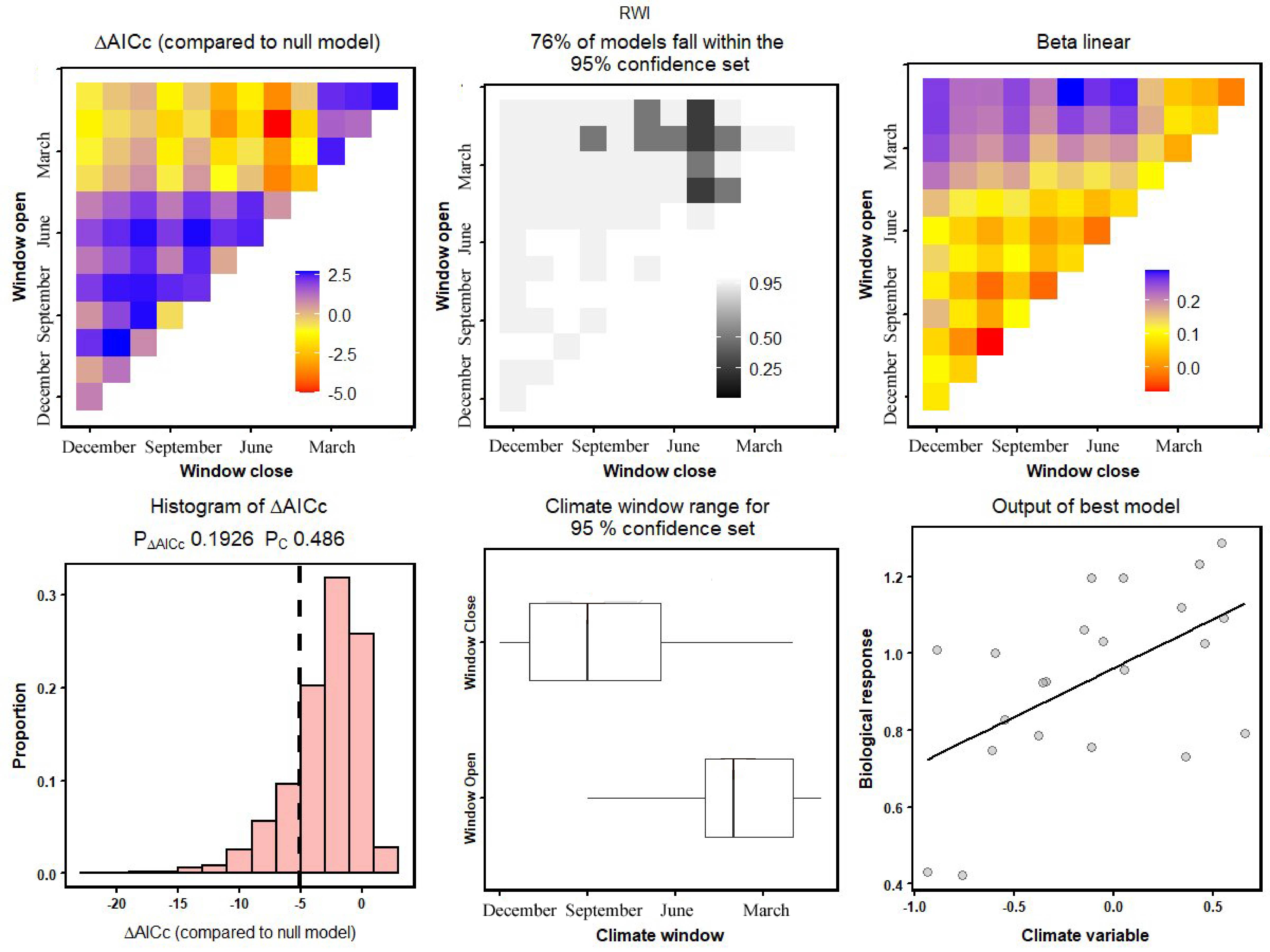


**Fig. S5.** Response correlation of TPX ring-width chronology and month resolved SPEI: “*climwin*” output panels obtained by calculating with K-fold cross-validation and randomization method (in the histogram of ΔAICc panel the vertical dashed line shows the ΔAICc of the best model fitted on the observed data).

**S6 Interannual variability in the SPEI_01 of the nearest meteorological stations**


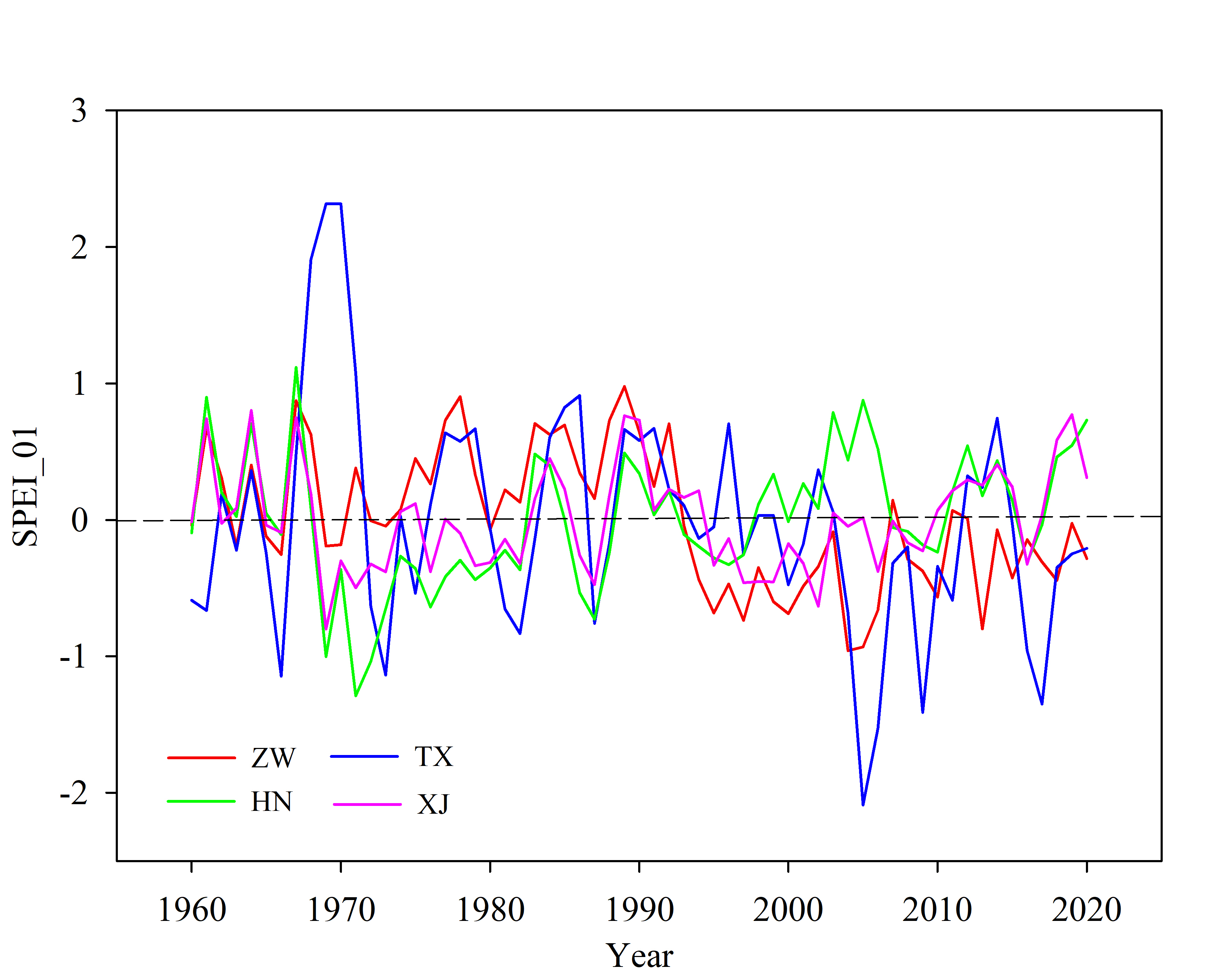


**Fig. S6.** Interannual variability in the SPEI_01 of the nearest meteorological stations

**S7 Influence of branch coppicing for soil water content at XZJ site in September, 2020**


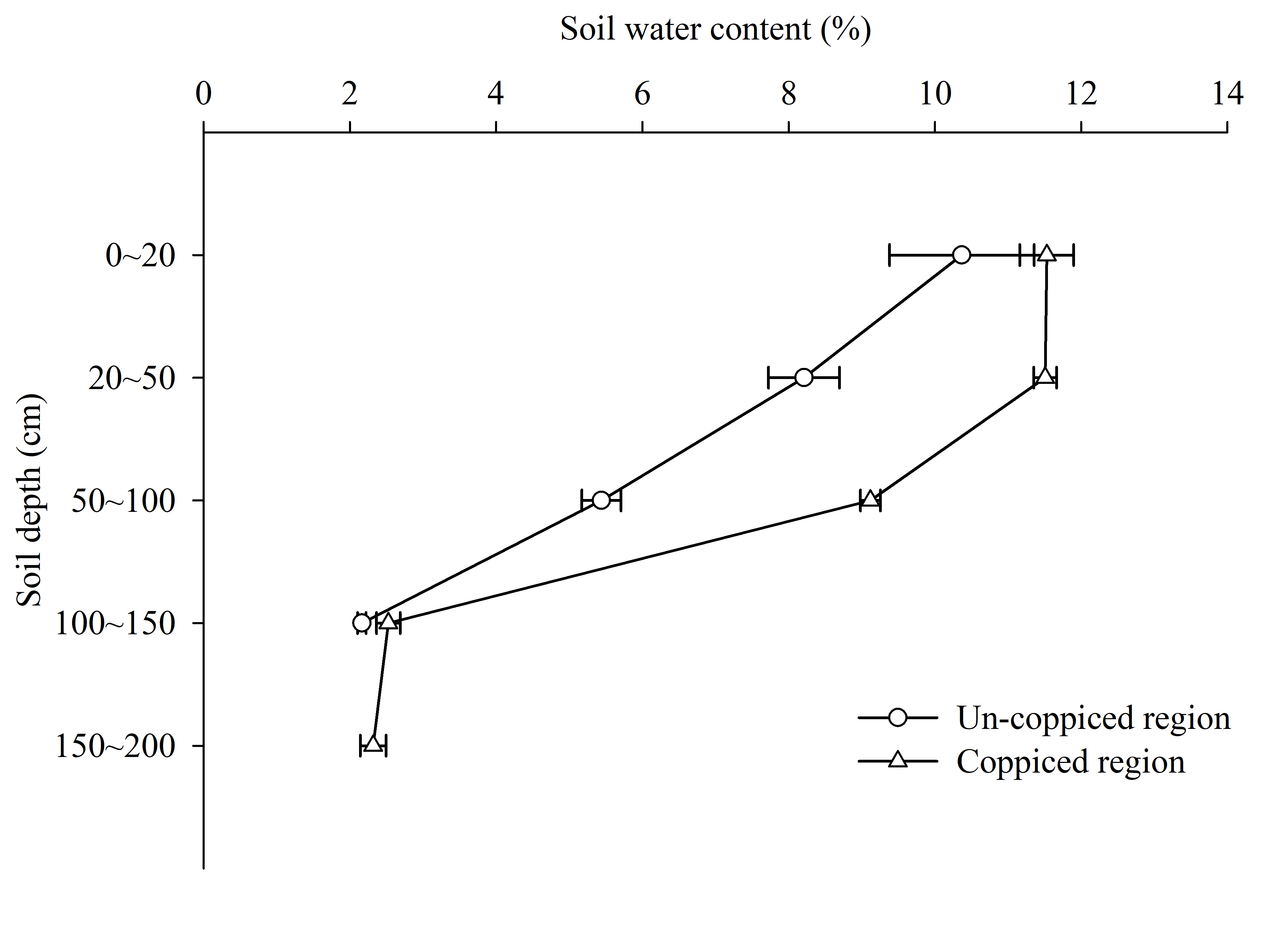


**Fig. S7.** Influence of branch coppicing for soil water content at XZJ site in September, 2020

1. ^*^ Corresponding author at: Key Laboratory of Ecohydrology of Inland River Basin, Northwest Institute of Eco-Environment and Resources, Chinese Academy of Sciences, China.

   *E-mail address*: [xiaosc@lzb.ac.cn](mailto:xiaosc@lzb.ac.cn) (S.-C. Xiao) [↑](#footnote-ref-2)
